# Solution of Camphor in Erysipelas

**Published:** 1875-03

**Authors:** 


SOLUTION OF CAMPHOR IN ERYSIPELAS.—In the *Gazetta Medica da Bahia* it is said that a few drops of a solution of equal parts of gum camphor and ether, applied from time to time to an erysipelatous surface will, in the majority of cases, affect a cure.—*New Remedies*, Jan., 1875, p. 15.

